# SOX2 interferes with the function of CDX2 in bile acid-induced gastric intestinal metaplasia

**DOI:** 10.1186/s12935-019-0739-8

**Published:** 2019-01-31

**Authors:** Ting Yuan, Zhen Ni, Chuan Han, Yali Min, Nina Sun, Caifang Liu, Miao Shi, Wenquan Lu, Na Wang, Feng Du, Qiong Wu, Ning Xie, Yongquan Shi

**Affiliations:** 1 State key Laboratory of Cancer Biology, National Clinical Research Center for Digestive Diseases and Xijing Hospital of Digestive Diseases, Fourth Military Medical University, Xi’an, 710032 China; 2 The 150 Hospital of the People’s Liberation Army, Luoyang, 471000 China; 3 Department of Gastroenterology, The General Hospital of Western Theater Command, Chengdu, 610083 China; 4 Rocket Army Emei Sanatorium, Emei, 614200 China; 5 Department of Gastroenterology, The Second Affiliated Hospital of Xi’an Medical University, Xi’an, 710032 China; 6 College of Postgraduates, Xi’an Medical University, Xi’an, 710032 China; 7 Department of Gastroenterology, First Affiliated Hospital, Zhengzhou University, Zhengzhou, 450000 China; 8 Department of Gastroenterology, Second Affiliated Hospital of Xi’an Jiaotong University, Xi’an, 710032 China

**Keywords:** Bile acid, CDX2, Intestinal metaplasia, miR-21, SOX2

## Abstract

**Background:**

Intestinal metaplasia (IM) is a premalignant lesion associated with gastric cancer. Both animal and clinical studies have revealed that bile acid reflux and subsequent chronic inflammation are key causal factors of IM. Previous studies indicated that SOX2, the key transcription factor in gastric differentiation, was downregulated during IM development while CDX2, the pivotal intestine-specific transcription factor was upregulated significantly. However, it remains unclear whether the downregulation of SOX2 promotes gastric IM emergence or is merely a concomitant phenomenon. In addition, the underlying mechanisms of SOX2 downregulation during IM development are unclear.

**Methods:**

Gastric cell lines were treated with deoxycholic acid (DCA) in a dose-dependent manner. The expression of CDX2 and miR-21 in gastric tissue microarray were detected by immunohistochemistry and in situ hybridization. Coimmunoprecipitation and immunofluorescence were performed to ascertain the interaction of SOX2 and CDX2. Luciferase reporter assays were used to detect the transcriptional activity of CDX2, and confirm miR-21 binding to SOX2 3′-UTR. The protein level of SOX2, CDX2 and downstream IM-specific genes were investigated using western blotting. mRNA level of miR-21, SOX2, CDX2 and downstream IM-specific genes were detected by qRT-PCR.

**Results:**

Bile acid treatment could suppress SOX2 expression and simultaneously induce expression of CDX2 in gastric cell lines. Furthermore, we demonstrated that SOX2 overexpression could significantly inhibit bile acid- and exogenous CDX2-induced IM-specific gene expression, including KLF4, cadherin 17 and HNF4α expression. In contrast, SOX2 knockdown had the opposite effect. A dual-luciferase reporter assay demonstrated that SOX2 overexpression could significantly suppress CDX2 transcriptional activity in HEK293T cells. CDX2 and SOX2 could form protein complexes in the nucleus. In addition, bile acid induced the expression of miR-21. The inhibition of SOX2 in bile acid-treated gastric cell lines was rescued by miR-21 knockdown.

**Conclusions:**

These findings suggested that SOX2 can interfere with the transcriptional activity of CDX2 in bile acid-induced IM and that miR-21 might play a key role in this process, which shed new lights in the prevention of gastric cancer.

**Electronic supplementary material:**

The online version of this article (10.1186/s12935-019-0739-8) contains supplementary material, which is available to authorized users.

## Background

Gastric cancer is the second most common malignancy and the second leading cause of cancer-related death in China [1]. The development of gastric cancer, especially intestinal type, usually occurs through chronic gastric inflammation, atrophic gastritis and intestinal metaplasia (IM) [2]. In general, *Helicobacter pylori* (Hp) is considered the most important etiological factor in both the precursor event and subsequent gastric cancer development [3, 4]. However, a number of studies have shown that Hp eradication cannot reverse IM progression [5, 6]. Hence, we speculate that predisposing factors other than Hp infection may play significant roles in IM development and progression. Consistent with this idea, a previous study demonstrated that prolonged bile reflux is a crucial factor in intestinal transformation at the gastroesophageal junction [7]. Patients with high bile acid concentrations in gastric juice manifest more extensive and more severe IM [8].

As a homeobox transcription factor, CDX2 is essential for intestinal cell growth and differentiation and is mainly expressed in the colon and small intestine [9]. Previous studies have reported that CDX2 transgenic mice can develop IM and gastric cancer, highlighting CDX2 as a molecular trigger in IM and carcinogenesis [10, 11]. Previous studies from other groups and our group also indicated that bile acid could induce CDX2- and IM-related gene expression in vitro [12, 13]. Nevertheless, the exact molecular network that promotes CDX2 upregulation in IM development is still not completely understood.

In contrast to CDX2, SOX2 is a member of the SRY-related HMG Box (SOX) family and was identified as a critical transcription factor for esophageal and gastric differentiation [14]. It has been noted that SOX2 is a tumor suppressor that inhibits cell proliferation and metastasis by regulating PTEN in gastric cancer [15]. A number of studies have found a converse expression pattern between SOX2 and CDX2 in IM tissue [16]. However, the relationship between SOX2 and CDX2 is still controversial. It remains unclear whether the downregulation of SOX2 can promote CDX2 expression and subsequent gastric IM development or is a concomitant phenomenon. In addition, the molecular mechanism by which SOX2 downregulation is involved in IM remains largely unknown.

MicroRNAs (miRNAs) are endogenously expressed small noncoding RNAs that play important gene-regulatory roles through binding to the 3′-untranslated regions (3′-UTRs) of target mRNAs [17]. To date, a number of studies have indicated that miRNAs are involved in the pathogenesis of many types of cancer, including gastric cancer [18, 19]. Among these miRNAs, miR-21 is one of the most common and highly upregulated miRNAs in gastric cancer and preneoplasia lesions [20–22]. The expression of miR-21 is significantly related to tumor size, metastasis and later-stage disease in patients with gastric cancer [23]. Based on these findings, we hypothesized that miR-21 may also mediate the phenotypic changes of gastric cells in the progression of IM.

In this study, we aimed to explore the relationship between SOX2 and CDX2 and the role of miR-21 in bile acid-treated gastric cell lines. The results showed that SOX2 could significantly suppress the intestine-specific markers KLF4, cadherin 17 and HNF4α in CDX2-overexpressing or bile acid-treated gastric cell lines. Additionally, luciferase reporter assays indicated that SOX2 could strongly suppress the transcriptional activity of CDX2. Coimmunoprecipitation experiments proved that SOX2 and CDX2 could form protein complexes in the AGS cell line. Moreover, miR-21, which was induced by bile acid, inhibited the expression of SOX2 by directly binding its 3′-UTR. In addition, inhibition of SOX2 in bile acid-treated gastric cell lines could be rescued by miR-21 knockdown. Our research provided new insights into the transformation of gastric IM after chronic bile acid reflux and SOX2 regulation at the miRNA level which shed new lights in the early prevention of gastric cancer.

## Methods

### Cell lines and drug treatment

The human normal gastric epithelial cell line GES-1 and the human gastric cancer cell lines AGS, AZ-521, BGC823, SGC7901 and MKN45 (purchased from ATCC Manassas, US) were cultured in RPMI-1640 medium (Gibco, CA, US) supplemented with 10% fetal bovine serum (Biological Industries, Beit Haemek, Israel), 100 mg/ml streptomycin and 100 U/ml penicillin. All cells were incubated at 37 °C in a humidified incubator containing 5% CO_2_. Deoxycholic acid (DCA) (MedChem Express, US) was dissolved in dimethyl sulfoxide (DMSO). After 24 h of starvation, the cells were exposed to 50–200 µM DCA for 24 h. These doses of DCA were chosen from previous in vivo and in vitro studies [24, 25].

### Tissue microarray and human paired IM samples

Paraffin-embedded consecutive gastric tissue slides (ST8017a, ST806, and IC00011c), which contained 141 cases of IM and 62 cases of normal gastric tissue, were purchased from Alenabio (Xi’an, China). Of these 141 IM patients, 121 were male (85.8%). The median age of all 141 IM patients was 58 years (range 32–85 years). Of these 62 normal patients, 38 were male (61.3%). The median age of all 62 normal patients was 62 years (range 36–82 years). Patients with mild intestinal metaplasia was 58 (41.1%). Patients with moderate intestinal metaplasia was 42 (29.8%). And patients with severe intestinal metaplasia was 41 (29.1%). All 203 cases were HP negative when these specimens were taken.

Paired IM specimens from eight individuals were obtained from the Xijing Hospital of Digestive Diseases. The pathological status of these specimens was provided by the Department of Pathology. All patients were HP negative to eliminate the impact of HP infection. This study was approved by the hospital’s Protection of Human Subjects Committee.

### RNA extraction and qRT-PCR

Total RNA was extracted from cultured cells by QIAzol Lysis reagent (QIAGEN, Hilden, Germany) and then purified using the miRNeasy Mini kit (QIAGEN). Reverse transcription PCR (qRT-PCR) was performed using the PrimeScript RT reagent kit (TaKaRa, Dalian, China) and the Mir-X miRNA First-Strand Synthesis Kit (TaKaRa). Quantitative real-time PCR was performed using TB Green Premix Ex Taq II (TaKaRa) and measured in a LightCycler 480 system (Roche, Basel, Switzerland). U6 and GAPDH were used as the internal controls for miRNA and mRNA assays, respectively. Each reaction was performed in triplicate. All of the primers are listed in Additional file 1: Table S1.

### Protein extraction and western blotting

Radioimmunoprecipitation assay (RIPA) buffer (Beyotime, Haimen, China) supplemented with protease and phosphatase inhibitors was used to prepare whole-cell lysates, and western blotting was performed as described previously [26]. The primary antibodies used were specific for SOX2 (Abcam, Cambridge, UK), CDX2 (Cell Signaling Technology, MA, USA), KLF4 (Cell Signaling Technology), cadherin 17 (Cell Signaling Technology), HNF4α (Abcam) and β-actin (ABclonal Technology, Wuhan, China).

### Immunofluorescence (IF)

IF staining for SOX2 and CDX2 was performed in AGS and AZ-521 cells. The primary antibodies used for IF were a rabbit anti-human CDX2 antibody (Cell Signaling Technology) and a mouse anti-human SOX2 antibody (Abcam).

### Immunohistochemistry (IHC) and in situ hybridization (ISH)

IHC staining was performed on tissue microarray chips (Alenabio) using an anti-CDX2 antibody (Cell Signaling Technology) following the manufacturer’s instructions. ISH was performed on the same tissue microarray chips (Alenabio) using an miR-21 probe from Exiqon (miRCURY LNA detection probe, 5′- and 3′-digoxigenin (DIG) labeled) according to the manufacturer’s instructions. The probe was detected using a DIG antibody (Abcam), LSAB2 System HRP (Dako Denmark A/S, Glostrup, Denmark), and the liquid DAB Substrate Chromogen System (Dako).

The IHC and ISH results were independently scored by two observers. The scoring was based on the percentage of positive cells and the intensity of staining. The percentage of positive cells was divided into four grades as follows: < 1% (0), 1–25% (1), 26–50% (2), 51–75% (3) and > 75% (4). Staining intensity was scored as follows: negative (0), weak (1), moderate (2) and strong (3). The histological score (H-score) for each sample was determined by the following formula: H-score = percentage score × intensity score. An overall score of 0–12 was calculated and graded as negative (score: 0), weak (score: 1–4), moderate (score: 5–8) or strong (score: 9–12).

### Oligonucleotide and plasmid transfection and lentiviral infection of target cells

An miR-21 mimic, an antagomir and their negative controls were purchased from RiboBio (Guangzhou, China). The SOX2 siRNA and its negative control oligonucleotides were purchased from GenePharma (Shanghai, China). Target cells were transfected with miR-21 mimics or antagomirs and the corresponding negative controls using the Lipofectamine RNAiMAX reagent (Invitrogen, CA, USA) according to the manufacturer’s protocol. The Lipofectamine RNAiMAX reagent (Invitrogen) was also used to transfect SOX2 siRNA into target cells. Cells cultured at 37 °C in Opti-MEM (Gibco) were collected after 48 h of transfection.

LV-SOX2 and LV-CDX2 were custom designed and provided by Genechem Co. Ltd. (Shanghai, China). Target cells were infected with the lentiviral LV-SOX2 and LV-CDX2 pool and selected with 2 μg/ml puromycin for 7 days after 48 h of transfection.

### Luciferase reporter assays

To detect the transcriptional activity of CDX2, a CDX2 transcriptional response element (TRE) was designed using the JASPAR database and inserted into the GV148 vector (sequence presented in Additional file 2: Figure S1f). HEK293T cells were cultured in 24-well plates and cotransfected with the SOX2 overexpression plasmid, the CDX2 overexpression plasmid and the CDX2 TRE plasmid using X-tremeGENE HP DNA Transfection Reagent (Roche). Cells were harvested and lysed for luciferase assays using a Dual-Luciferase Reporter Assay System (Promega, WI, USA) according to the manufacturer’s protocol after 48 h of transfection. Renilla and firefly luciferase activities were measured, and the luciferase score was calculated. Independent experiments were performed in triplicate for each condition.

For 3′-UTR reporter assays, HEK293T cells cultured in 24-well plates were transfected with miR-21 mimic, miR-21 inhibitor, or their negative controls and the indicated psiCHECK-2 plasmids using Lipofectamine 2000 (Thermo-Fisher, Lafayette, CO, USA). After 48 h of transfection, the cells were harvested and lysed for luciferase assays using a dual-luciferase reporter assay system. Firefly luciferase activity normalized to Renilla luciferase was used as an internal control. Three independent transfection experiments were performed for each plasmid construct.

### Coimmunoprecipitation (Co-IP)

Pierce IP Lysis Buffer (Thermo Scientific HyClone, Beijing, China) was used for cell lysates. Co-IP experiments were then performed using the PureProteome™ Protein G Magnetic Bead System (LSKMAGG02, Millipore, Schwalbach/Ts., Germany) according to the manufacturer’s instructions. Normal rabbit lgG (Millipore) was used as a negative control antibody. A rabbit anti-Sox2 mAb (Cell Signaling Technology) and a rabbit anti-CDX2 mAb (Cell Signaling Technology) were used as the capture antibodies according to the antibody manufacturer’s recommendations. Western blotting was used to analyze the immunoprecipitate eluted from the protein G magnetic beads.

### Statistical analysis

All statistical data were analyzed with SPSS software (version 19.0, SPSS Inc., Chicago, IL, USA). Continuous data are presented as the mean ± SD. Comparisons between two groups were performed using unpaired Student’s *t* test, and comparisons between multiple groups were performed using one-way analysis of variance (ANOVA) with the Bonferroni post hoc test. Frequencies of categorical variables were compared using the χ^2^ test. P < 0.05 was considered statistically significant.

## Results

### Bile acids induced CDX2 while suppressing SOX2 in gastric cell lines

**Fig. 1:**
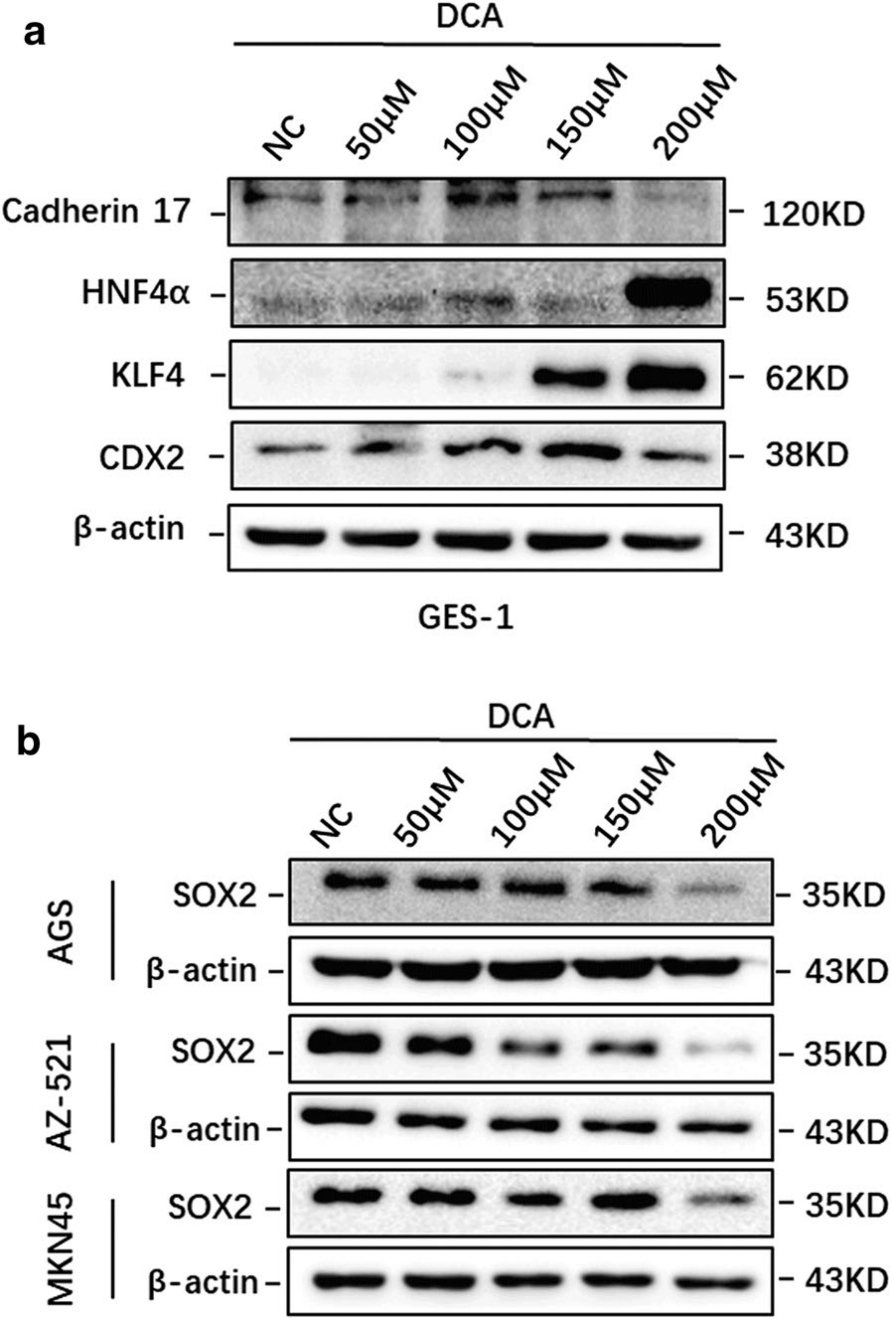
Bile acid induced CDX2 and intestinal markers while suppressing SOX2 in gastric cell lines. **a** The expression of CDX2, KLF4, HNF4α, and cadherin 17 was increased upon stimulation with DCA in a dose-dependent manner. **b** DCA treatment inhibited the expression of SOX2 in AGS, AZ-521 and MKN45 cells. Dose: 50, 100, 150 and 200 μM for 24 h

A previous study explored the effects of bile acid on esophageal cells in vitro and demonstrated that DCA at neutral pH could activate NF-kB and IL-8 expression at specific concentrations for 24 h [25]. In addition, NF-kB was reported to promote CDX2 expression transcriptionally [27, 28]. As a human normal gastric epithelial cell line, GES-1 cells express a trace amount of endogenous CDX2 (Additional file 2: Figure S1a, c). Hence, we first treated GES-1 cells with DCA, a type of unconjugated bile acid, at different doses for 24 h. The expression of CDX2 and several intestinal markers (KLF4, cadherin 17 and HNF4α) was examined by western blotting. The results indicated that CDX2 and intestinal markers were substantially upregulated after 24 h of DCA treatment at 100–200 μM (Fig. 1a). As SOX2 was also absent in GES-1 cells (Additional file 2: Figure S1b, c), we treated AGS, AZ-521 and MKN45 cells with different doses of DCA for 24 h. Western blotting results showed that DCA could strongly suppress the expression of SOX2 at a 200 μM concentration (Fig. 1b). Taken together, these results suggest that DCA could increase the expression of CDX2 and downstream intestine-specific markers but suppress the expression of SOX2 in gastric cell lines.

### Upregulation of CDX2 was sufficient to drive the activation of the intestinal phenotype

**Fig. 2:**
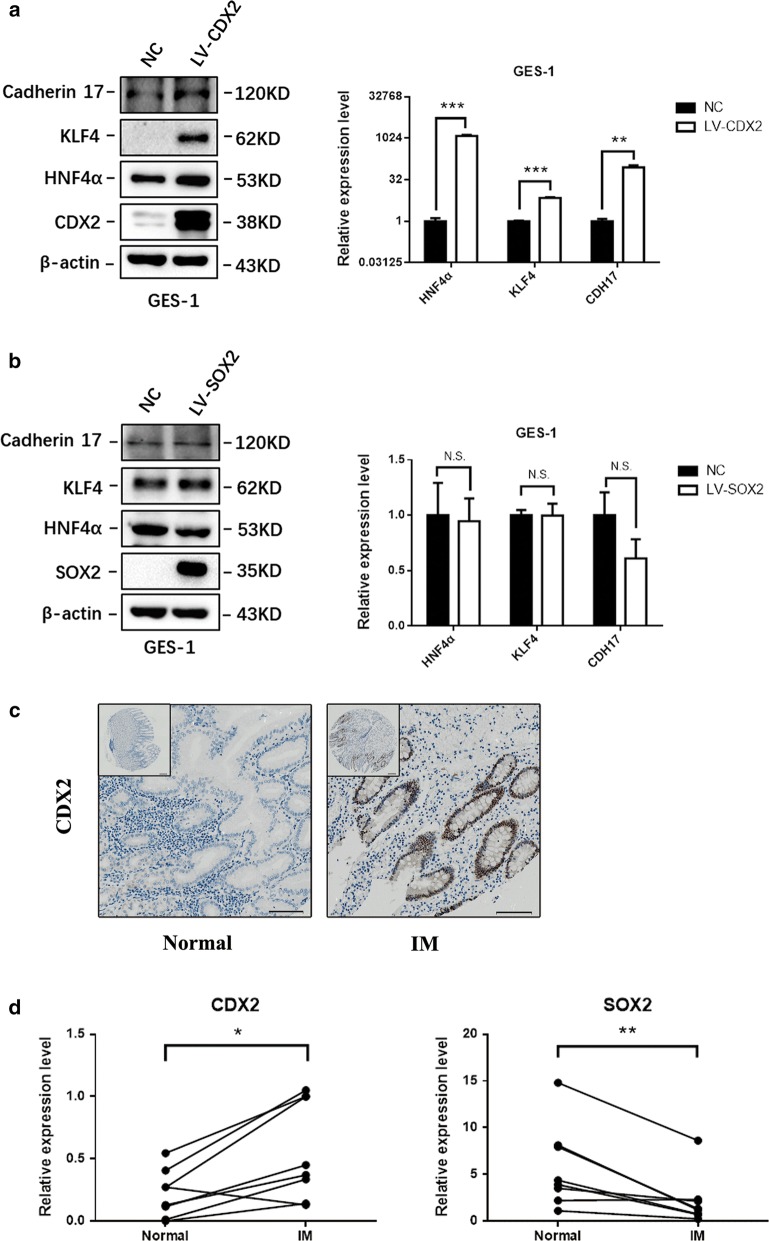
Upregulation of CDX2 was sufficient to drive the activation of the intestinal phenotype. **a** CDX2 increased the expression of KLF4, HNF4α, and cadherin 17 at the protein and mRNA levels in GES-1 cells. **b** In the normal gastric cell line GES-1, ectopic expression of SOX2 failed to regulate the expression of KLF4, HNF4α, and cadherin 17 at both the protein and mRNA levels. **c** The expression level of CDX2 in normal gastric tissues and gastric intestinal metaplasia tissues; scale bars: 200 μm (top) and 100 μm (bottom). **d** qRT-PCR of CDX2 and SOX2 levels in 8 pairs of matched human IM specimens. Each symbol represents mean value of an individual patient. *P < 0.05; **P < 0.01; *NS* not significant (P > 0.05)

It has been reported that KLF4, cadherin 17 and HNF4α are closely related to IM in the esophagus and stomach. In addition, all three IM-specific factors can be directly regulated by the transcription factor CDX2 [29–32]. To better assess the significance of CDX2 and SOX2 in IM, we first infected GES-1 cells with SOX2 and CDX2 overexpression constructs, respectively. Both western blotting and qRT-PCR results indicated that CDX2 could significantly upregulate KLF4, cadherin 17 and HNF4α, while there was no direct regulatory relationship between SOX2 and the three intestine-specific factors (Fig. 2a, b).

The expression of CDX2 in IM tissue and normal gastric epithelial tissue was investigated by IHC in three tissue microarray chips that contain 141 cases of IM and 62 cases of normal gastric tissue. Compared with that in normal tissues, the level of CDX2 was increased in IM tissues. The expression of CDX2 was diffuse in 76 (53.9%) of 141 IM cases. Only 9 (14.5%) of 62 normal gastric cases showed CDX2 staining. All CDX2-positive cases showed nuclear staining, and we did not observe cytoplasmic or membranous staining for CDX2 in any of the specimens examined (Fig. 2c). After statistical analysis, the results indicated a strong correlation between the upregulation of CDX2 and IM status (Table 1) (P < 0.01).

**Table 1:** CDX2 expression in IM and normal gastric tissues

	CDX2 expression level			
	Negative (−)	Weak (+)	Strong (++ or +++)	P-values
IM (%)	65/141 (46.1)	61/141 (43.3)	15/141 (10.6)	–
Normal (%)	53/62 (85.5)	8/62 (12.9)	1/62 (1.6)	< 0.01

To further examine the expression of SOX2 and CDX2 in human samples with qRT-PCR, we obtained paired IM specimens from 8 individuals in our hospital. All specimens we collected were HP negative to eliminate the impact of HP infection. qRT-PCR shown that CDX2 was overexpressed in IM samples (P < 0.05) while SOX2 was downregulated in IM samples (P < 0.01) (Fig. 2d).

### SOX2 interfered with CDX2 transcriptional activity in both exogenous CDX2-overexpressing and bile acid-treated gastric cell lines

**Fig. 3:**
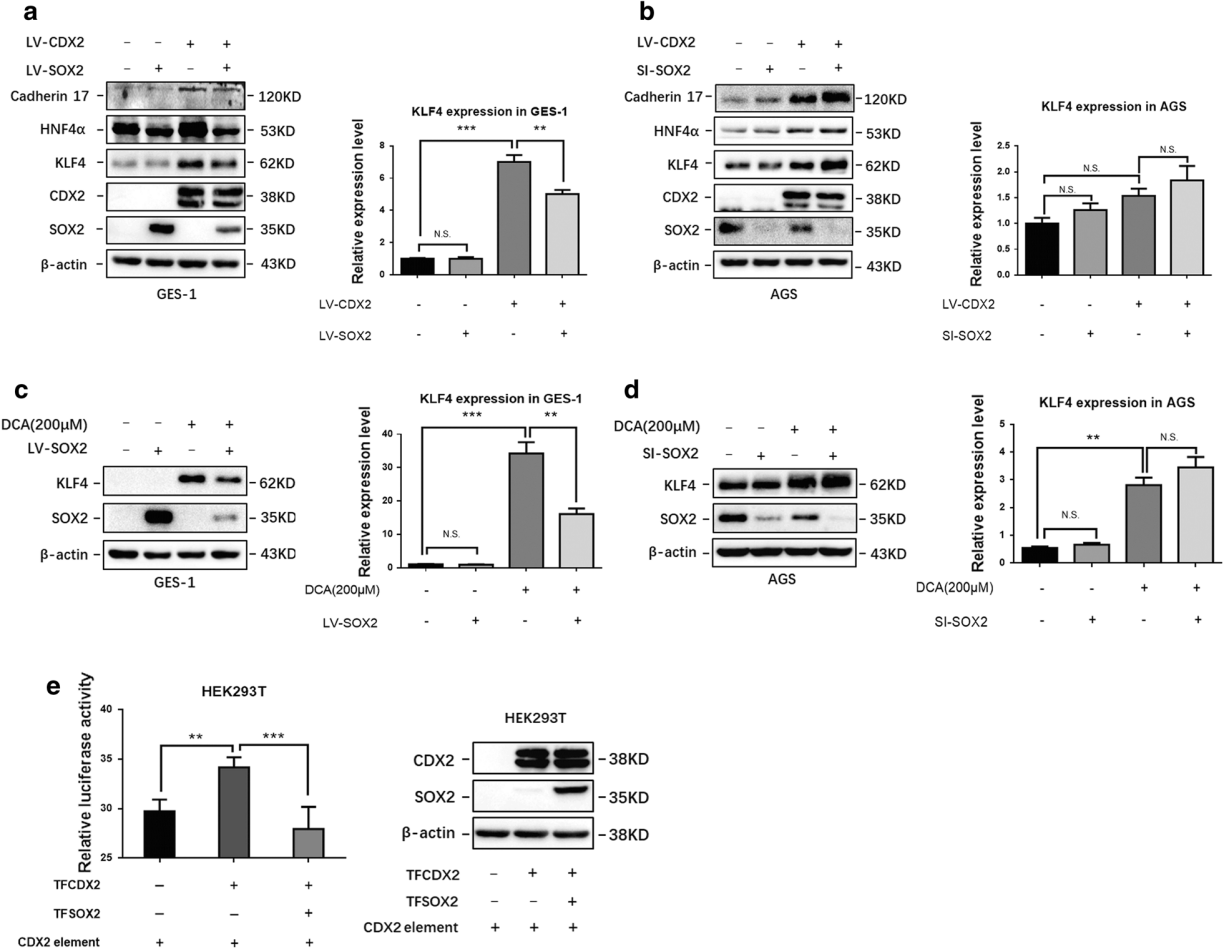
SOX2 interfered with CDX2 transcriptional activity in CDX2-overexpressing and bile acid-treated gastric cell lines. **a**, **b** SOX2 inhibited KLF4, HNF4α, and cadherin 17 in CDX2-overexpressing GES-1 and AGS cells. **c**, **d** SOX2 inhibited the expression of KLF4 in DCA-treated GES-1 and AGS cells. Dosage: 200 μM for 24 h. **e** CDX2 transcriptional response element (TRE) reporter plasmids were cotransfected with a SOX2-overexpressing plasmid and a CDX2-overexpressing plasmid or their negative controls (NCs) in HEK293T cells. *P < 0.05; **P < 0.01; *NS* not significant (P > 0.05)

To further explore the relationship between the CDX2 and SOX2 transcription factors and IM, we next coinfected GES-1 cells with SOX2- and CDX2-overexpressing lentiviruses. Surprisingly, the western blotting results indicated that compared with that in the control group, the expression of KLF4, HNF4α and cadherin 17 was induced by CDX2 and reduced by SOX2 in GES-1 cells. Furthermore, qRT-PCR also confirmed that SOX2 suppressed KLF4 expression in CDX2 lentivirus-infected GES-1 cells (Fig. 3a). In addition, knockdown of SOX2 in AGS cells that expressed endogenous SOX2 had the opposite effect (Fig. 3b). To further examine the function of SOX2 in bile acid-induced gastric cells, SOX2-overexpressing GES-1 cells and SOX2 knockdown AGS cells were treated with 200 μM DCA for 24 h before harvesting for further experiments. Western blotting and qRT-PCR showed that DCA-induced KLF4 expression was decreased in SOX2-overexpressing GES-1 cells compared with that in the control group (Fig. 3c). As expected, the expression of KLF4 was further increased in SOX2 knockdown AGS cells at the protein level (Fig. 3d).

To assess the effect of SOX2 on CDX2 transcriptional activity, a CDX2 TRE was inserted into the GV148 vector, and a SOX2-overexpressing plasmid and a CDX2-overexpressing plasmid were cotransfected into HEK293T cells. Compared with the negative control, ectopic expression of CDX2 strongly increased the luciferase activities of the CDX2 TRE reporter constructs. Furthermore, ectopic expression of SOX2 significantly attenuated the activation of CDX2 TRE induced by CDX2. These results clearly indicated that SOX2 could strongly suppress the transcriptional activity of CDX2 (Fig. 3e).

### SOX2 and CDX2 interacted with each other at the protein level

**Fig. 4:**
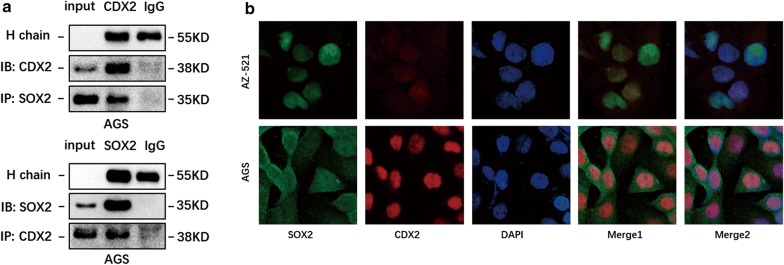
SOX2 and CDX2 formed protein complexes in the nucleus. **a** Positive result of CDX2 and SOX2 cross-linking Co-IP assays. **b** Immunofluorescence results showed that SOX2 and CDX2 colocalize in the nucleus in AGS and AZ-521 cells

Although several studies have investigated the relationship between SOX2 and CDX2, the mutual interaction of CDX2 and SOX2 in gastric IM progression is still unclear [16, 33, 34]. The above results indicated that SOX2 could significantly interfere with CDX2 transcriptional activity. Therefore, we proposed that these two transcription factors may interact with each other at the protein level. Co-IP analysis revealed that endogenous SOX2 and CDX2 could form protein complexes in AGS cells (Fig. 4a). Furthermore, IF staining for SOX2 and CDX2 was performed in AGS and AZ-521 cells. The IF results proved that SOX2 and CDX2 colocalized in the nucleus in AGS and AZ-521 cells (Fig. 4b).

### miR-21 was involved in bile acid-induced SOX2 downregulation

**Fig. 5:**
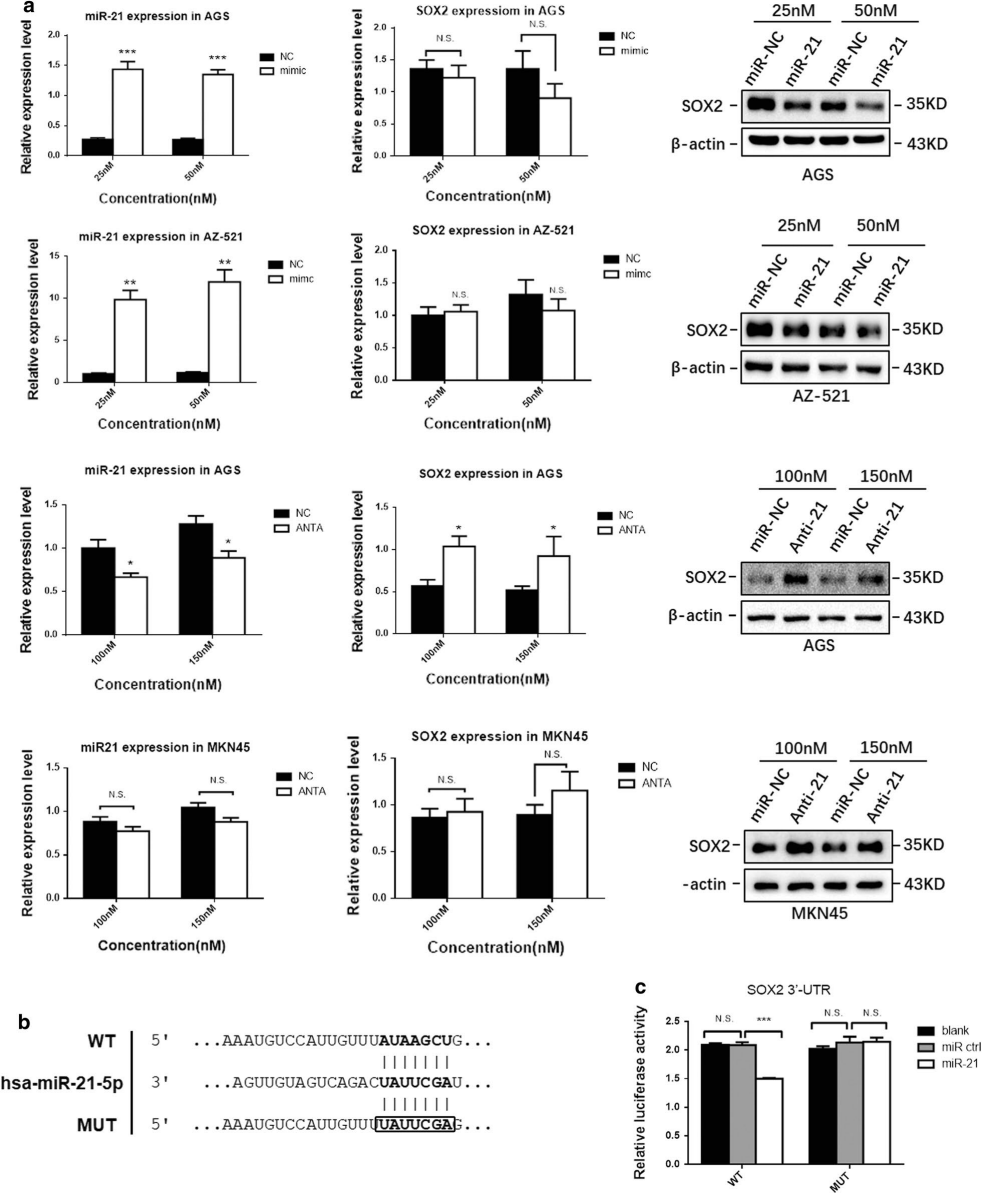
miR-21 downregulated SOX2 by directly binding its 3′-UTR. **a** Western blotting results showed that miR-21 inhibited the expression of SOX2 in gastric cell lines, while SOX2 mRNA levels were maintained in most groups. **b** Diagram of the SOX2 3′-UTR-containing reporter construct. Mutations were generated at predicted miR-21 binding sites located in the SOX2 3′-UTR. **c** Representative luciferase activity in HEK293T cells cotransfected with wild-type or mutated reporter plasmids and miR-ctrl or miR-21. *P < 0.05; **P < 0.01; *NS* not significant (P > 0.05)

miRNAs are important regulators of gastric cancer and IM. Among these miRNAs, miR-21 is one of the most common and highly upregulated miRNAs in gastric cancer and IM [21, 22]. A prior study indicated that miR-21 suppressed SOX2 expression in human mesenchymal stem cells (MSCs) and acted as a key determinant of MSC proliferation and differentiation [35]. As shown above, DCA significantly inhibited the expression of SOX2 at the protein level but not at the mRNA level (Additional file 2: Figure S1d, e). Thus, we presumed that miR-21 might posttranscriptionally repress SOX2 by targeting its 3′-UTR in gastric cells. To verify our hypothesis, AZ-521, AGS and MKN45 cells were transfected with miR-21 mimics or antagomirs. Western blotting results revealed that ectopic miR-21 expression reduced the protein levels of SOX2 in AGS and AZ-521 cells, while miR-21 knockdown increased SOX2 expression in AGS and MKN45 cells (Fig. 5a). qRT-PCR analysis showed that SOX2 mRNA levels were maintained in most groups transfected with miR-21 mimics or antagomirs (Fig. 5a).

To further investigate the regulatory relationship between miR-21 and SOX2, a dual-luciferase reporter assay was performed in HEK293T cells. Either the wild-type or mutant SOX2-3′-UTR was cloned downstream of a luciferase open reading frame, and firefly luciferase activity normalized to Renilla luciferase was used as an internal control (Fig. 5b). Overexpression of miR-21 inhibited the luciferase activity of the SOX2 3′-UTR reporter construct, whereas no effect was detected when mutations were introduced into its seed sequences (Fig. 5c).

Together, these experiments confirmed that miR-21 could suppress the expression of SOX2 by directly binding the SOX2 3′-UTR.

**Fig. 6:**
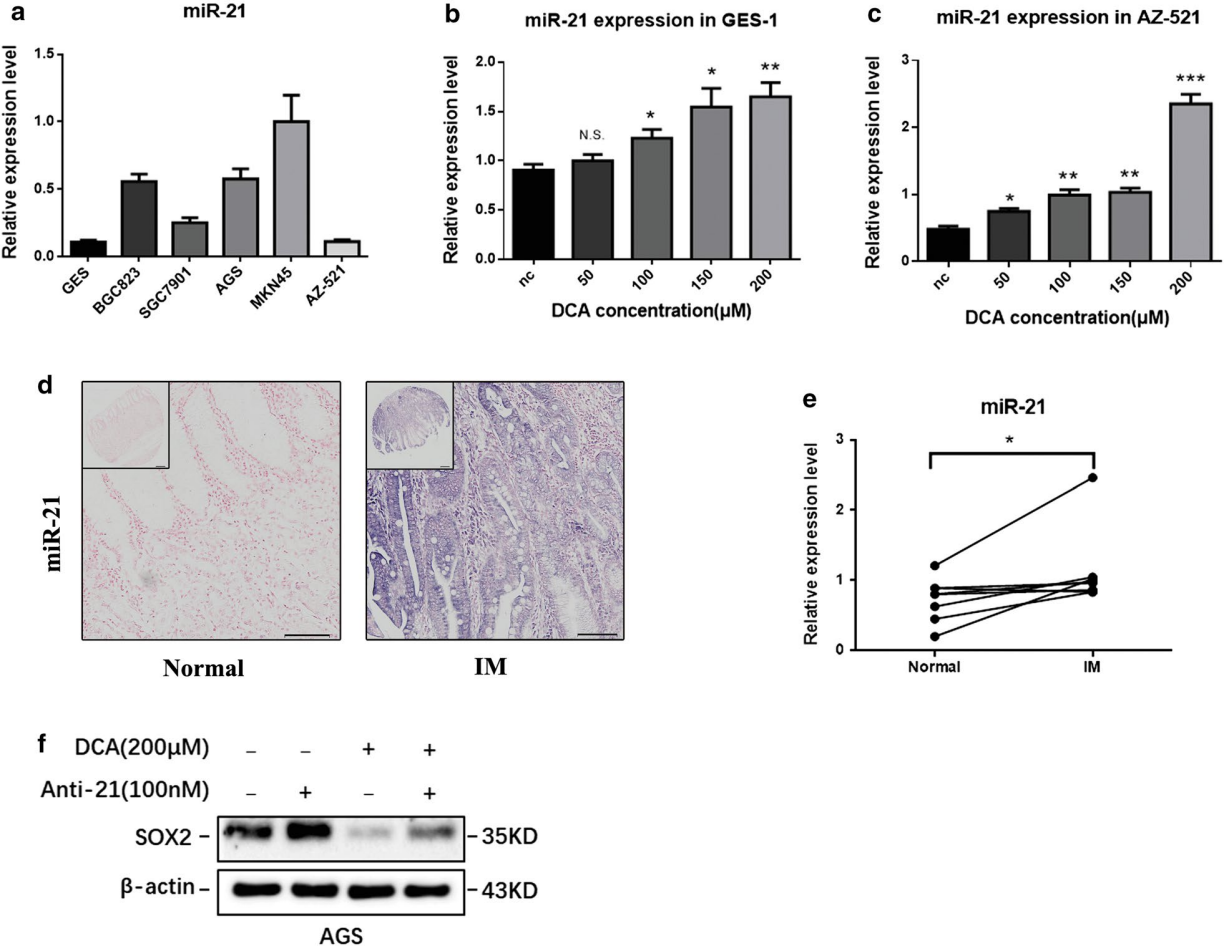
miR-21 was increased in bile acid-stimulated gastric cells and IM. **a** The expression of miR-21 in 6 gastric cell lines. **b**, **c** The expression of miR-21 was increased upon stimulation with DCA in a dose-dependent manner in GES-1 and AZ-521 cells. Dose: 50, 100, 150 and 200 μM for 24 h. **d** The expression level of miR-21 in normal gastric tissues and gastric intestinal metaplasia tissues; scale bars: 200 μm (top) and 100 μm (bottom). **e** qRT-PCR of miR-21 levels in 8 pairs of matched human IM specimens. Each symbol represents mean value of an individual patient. **f** Western blotting results indicated that the inhibition of SOX2 in bile acid-treated AGS cells was partially rescued in miR-21 knockdown AGS cells. *P < 0.05; **P < 0.01; *NS* not significant (P > 0.05)

Finally, to test whether miR-21 plays an important role in bile acid-induced IM, we examined the expression of miR-21 in bile acid-stimulated gastric cell lines, IM tissue microarray and paired IM specimens from 8 individuals in our hospital using qRT-PCR and ISH. Among the 6 gastric cell lines, MKN45 cells expressed the highest basal level of miR-21, while GES-1 and AZ-521 cells showed undetectable basal levels of miR-21 (Fig. 6a). After treatment with DCA for 24 h, the expression of miR-21 increased significantly in a dose-dependent manner in both GES-1 and AZ-521 cells (Fig. 6b, c). The expression of miR-21 was measured in three tissue microarray chips containing 141 cases of IM and 62 cases of normal gastric tissue by ISH (Fig. 6d). Our results indicated that miR-21 expression was significantly upregulated in IM tissue compared with that in normal gastric tissue (Table 2). qRT-PCR of miR-21 in 8 pairs of matched human IM specimens indicated the expression of miR-21 was higher in IM samples (P < 0.01) (Fig. 6e). Collectively, these results suggest that bile acids could increase miR-21 expression, which is upregulated in gastric IM tissues.

**Table 2:** miR-21 expression in IM and normal gastric tissues

	miR-21 expression level			
	Negative (−)	Weak (+)	Strong (++ or +++)	P-values
IM (%)	36/141 (25.5)	52/141 (36.9)	53/141 (37.6)	–
Normal (%)	30/62 (48.4)	18/62 (29.0)	14/62 (22.6)	< 0.01

To further explore the function of miR-21 in bile acid-induced gastric cells, miR-21 was knocked down in AGS cells by an antagomir. Then, 200 μM DCA was added for 24 h. Western blotting results indicated that the inhibition of SOX2 by bile acids in AGS cells was partially rescued by miR-21 knockdown (Fig. 6f).

## Discussion

As a precursor event of gastric cancer, IM can dramatically increase the incidence of gastric cancer in patients [36]. Generally, many etiological factors affect the progression of IM, including Hp infection, alcohol intake, cigarette smoking, radiation and bile acid reflux. However, the molecular mechanism of phenotypic transformation in stomach epithelium with IM remains largely elusive.

As a homeobox transcription factor, CDX2 is essential for intestinal differentiation and is mainly expressed in the intestinal epithelium. Previous studies in transgenic mice have demonstrated that ectopic CDX2 expression may be the early event that triggers the progression of IM in the gastric mucosa [10, 37]. Our results confirm that bile acid DCA can activate CDX2 and its downstream intestinal markers, including KLF4, HNF4α and cadherin 17, in gastric cell lines. Similar to our results, several studies on Barrett’s esophagus also indicated that CDX2 expression induced by bile acid reflux plays an important role in phenotypic change in the esophageal mucosa [27, 30]. In the present study, we found that CDX2 and several known CDX2 target genes could be induced by bile acid in gastric cell lines in a dose-dependent manner. Additionally, CDX2 expression was increased in IM tissue.

The transcription factor SOX2 is a member of the SRY-related HMG box family. SOX2 is involved in the regulation of embryonic development and in the determination of cell fate. In addition to being required for stem cell maintenance in the central nervous system, SOX2 also regulates gene expression in the stomach [38]. Aberrant SOX2 expression has been reported to be associated with various types of cancer, especially gastric cancer [39–41]. However, due to conflicting results, it is still unclear whether SOX2 is an oncogene or functions as a protective gene in cancer development. For instance, one study found that SOX2 was increased in gastric cancer, and tumors expressing high levels of SOX2 showed more extensive invasion, higher TNM stages, and worse prognoses [42]. However, another study found that compared with SOX2-positive gastric cancer, decreased SOX2 expression in gastric cancer was associated with an increased extent of tumor invasion, higher rates of lymph node metastasis and shorter survival [38]. In IM, a precancerous lesion of gastric cancer, most studies showed that SOX2 expression was absent [3, 16, 40]. Another study showed that SOX2 expression was maintained in CDX2-induced intestinal metaplastic mucosa even though the gastric phenotype was completely lost, which indicated that CDX2 expression is sufficient and the loss of SOX2 expression is not necessary for the progression of IM [34]. Interestingly, ectopic expression of SOX2 in the intestinal epithelium could convert the cell fate of already committed intestinal cells into stomach-like cells. CDX2 expression was maintained in SOX2-induced animals, while several known CDX2 target genes were clearly downregulated [33]. In the present study, we first demonstrated that the expression of SOX2 was decreased in bile acid-treated gastric cells, which might reveal a potential cause of SOX2 loss in IM tissue. To investigate the functions of SOX2 and CDX2 in IM, we coinfected SOX2- and CDX2-overexpressing lentiviruses into GES-1 cells. Our results showed that SOX2 inhibited CDX2-induced intestine-specific markers (KLF4, HNF4α and cadherin 17) in CDX2-overexpressing gastric cells, while there was no direct regulatory relationship between SOX2 and those markers in cells that lacked CDX2 expression. This result suggested that CDX2 is the molecular trigger in the progression of IM; in addition, although SOX2 was suppressed during this process, it might function as a protective factor by indirectly inhibiting intestine-specific factors. Our result was consistent with other researchers’ opinion that SOX2 might interfere with the function of CDX2 by suppressing its downstream DNA-binding capacity [33]. However, the molecular mechanism by which SOX2 interferes with the function of CDX2 is unclear. In this study, we demonstrated that SOX2 could strongly suppress the transcriptional effect of CDX2 on its genomic target sites using a dual-luciferase reporter assay. In addition, Co-IP was performed to investigate the relationship of these two factors at the protein level. Our results suggested that SOX2 and CDX2 could form protein complexes in the nucleus. Thus, the formation of a SOX2-CDX2 complex might be one of the most important mechanisms contributing to the subsequent suppression of intestine-specific markers in IM. Moreover, we found that bile acid-induced KLE4 was inhibited by overexpression of SOX2. Considering that bile acid is an exogenous stimulator of CDX2, this result verified our view that SOX2 inhibits the progression of intestinal phenotype conversion by interfering with the function of CDX2.

miRNAs are endogenously expressed small noncoding RNAs that play a vital role in the development of cancer and premalignant lesions [21–23]. Among those miRNAs, we focused on miR-21 because a recent study showed that miR-21 suppresses the expression of SOX2 in human MSCs [35]. Additionally, a previous study reported that bile acid appeared to accelerate the expression of the “oncomir” miR-21 in the laryngeal mucosa [43]. In the present study, to investigate the regulatory effect of miR-21 on SOX2 in gastric cells, miR-21 loss- and gain-of-function experiments and dual-luciferase reporter assays were performed to demonstrate that miR-21 regulates SOX2 by directly binding its 3′-UTR. Furthermore, qRT-PCR results suggested that miR-21 expression could be induced by bile acid in a dose-dependent manner. In addition, the ISH results showed that miR-21 expression was significantly increased in the IM sample compared with that in normal gastric tissue. Finally, we found that knockdown of miR-21 partially rescued the inhibition of SOX2 in bile acid-treated gastric cell lines. Taken together, these results suggest that miR-21 plays an important role in the progression of bile acid-induced IM by regulating the expression of SOX2.

## Conclusion

**Fig. 7:**
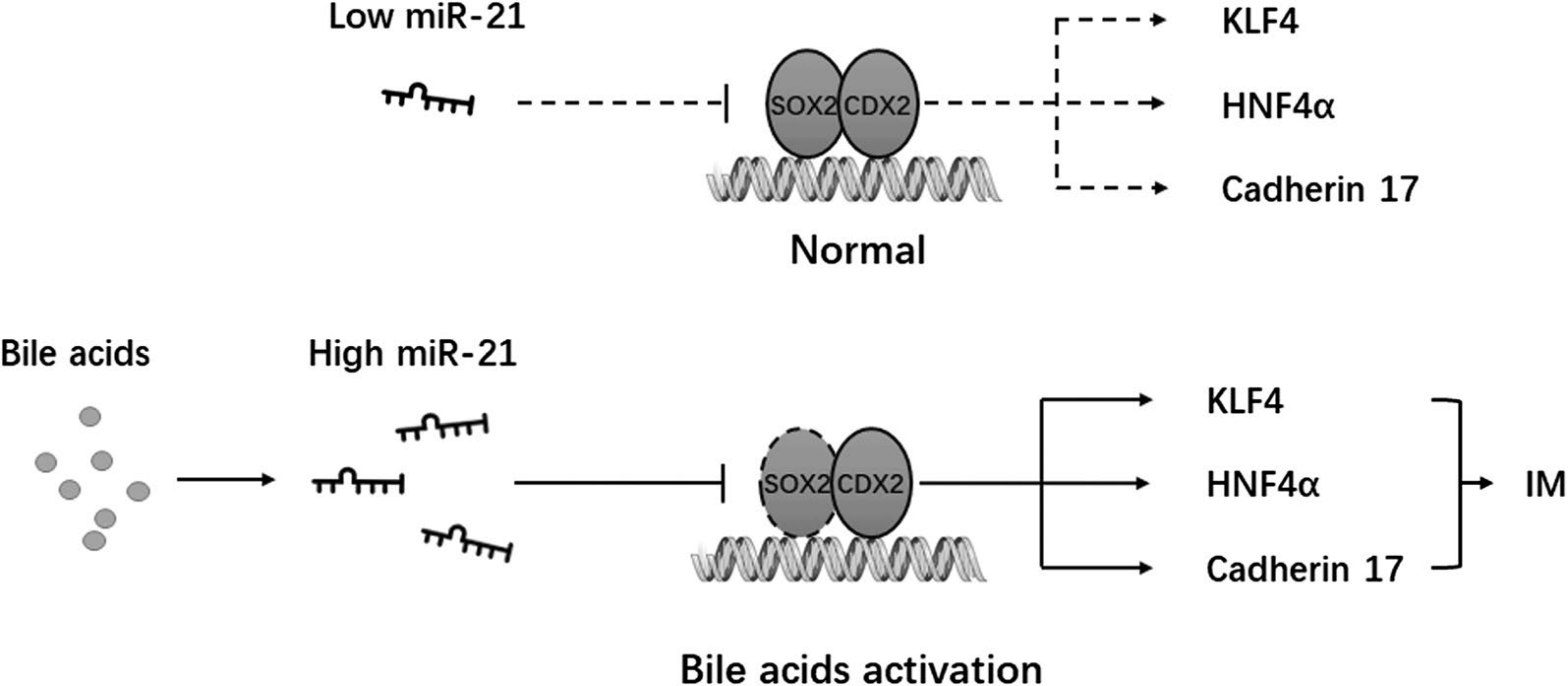
A schematic model of miR-21/SOX2/CDX2 pathway in gastric cells. Induced by specific concentrations of bile acid, miR-21 inhibits the expression of SOX2 and abrogates its suppression on the transcriptional activity of CDX2

In summary, our results demonstrated that bile acid induced the expression of CDX2 and its target genes but simultaneously inhibited SOX2 expression in gastric cells. Additionally, SOX2 could significantly suppress the function of CDX2 by formatting a SOX2-CDX2 protein complex. Moreover, miR-21, which can be induced by specific concentrations of bile acid, inhibits the expression of SOX2 by directly binding its 3′-UTR. The inhibition of SOX2 in bile acid-treated gastric cell lines can be rescued by knockdown of miR-21 (Fig. 7).

## Additional files

**Additional file 1: Table S1.** Sequences of PCR primers.

**Additional file 2: Figure S1.** (a, b) The expression of SOX2 and CDX2 at the mRNA level in gastric cell lines. (c) The expression of SOX2 and CDX2 at the protein level in gastric cell lines. (d, e) The expression of SOX2 at the mRNA level was not inhibited by DCA stimulation in MKN45 and AZ-521 cells. (f) Sequences of the CDX2 TRE with five sites predicted by the JASPAR database. (g) The mRNA level of CDX2, KLF4 in MKN45 and SOX2 expression in GES-1 after DCA treatment. (h) miR-21 level is not affected by CDX2 in GES-1 *P < 0.05; **P < 0.01; N.S., not significant (P > 0.05).

## Abbreviations

IM: intestinal metaplasia
DCA: deoxycholic acid
Hp: *Helicobacter pylori*
SOX: SRY-related HMG Box
3′-UTRs: 3′-untranslated regions
DMSO: dimethyl sulfoxide
RIPA: radioimmunoprecipitation assay
MSCs: mesenchymal stem cells
