# Supplementary material for: SOX2 interferes with the function of CDX2 in bile acid-induced gastric intestinal metaplasia
Source: Cancer Cell Int. 2019 Jan 31;19:24. doi: 10.1186/s12935-019-0739-8 (PMC6357452; doi:10.1186/s12935-019-0739-8)
Supplement: Supplementary file 1 — Additional file 1: Table S1. Sequences of PCR primers. [file 12935_2019_739_MOESM1_ESM.docx]

**Table S1.** Sequences of PCR primers

| PCR primers | |
| --- | --- |
| CDX2 | Forward: 5’- TTCACTACAGTCGCTACATCACCA -3’  Reverse: 5’- CTGCGGTTCTGAAACCAGATT -3’ |
| KLF4 | Forward: 5’- GTGCCCCGAATAACAGCTCA -3’  Reverse: 5’- TTCTCACCTGTGTGGGTTCG -3’ |
| SOX2 | Forward: 5’- CCAAGATGCACAACTCGGAGA -3’  Reverse: 5’- CCGGTATTTATAATCCGGGTGCT-3’ |
| HNF4α | Forward: 5’- GTTCAAGGACGTGCTGCTCCTA -3’  Reverse: 5’- AGGCATACTCATTGTCATCGATCTG -3’ |
| Cadherin 17 | Forward: 5’- AGCAGGTCACCAGACTGGGATAC -3’  Reverse: 5’- TCAGATGCTTGAGCACTTTCAACA -3’ |
| GAPDH | Forward: 5’-ATGTCGTGGAGTCTACTGGC-3’  Reverse: 5’-TGACCTTGCCCACAGCCTTG-3’ |
